# Transcriptome-Wide Identification and Quantification of Caffeoylquinic Acid Biosynthesis Pathway and Prediction of Its Putative BAHDs Gene Complex in *A. spathulifolius*

**DOI:** 10.3390/ijms22126333

**Published:** 2021-06-13

**Authors:** Sivagami-Jean Claude, Sunmi Park, Seon-Joo Park

**Affiliations:** Department of Life Sciences, Yeungnam University, Gyeongsan 38541, Korea; sivaynu@ynu.ac.kr (S.-J.C.); psm1128@yu.ac.kr (S.P.)

**Keywords:** phenylpropanoid pathway (PPP), 5-Caffeoylquinic acid (5-CQA or CQA), BAHD acyltransferases (BAHDs), hydroxycinnamoyl-coenzyme A: quinate hydroxycinnamoyl transferase (*HQT*), hydroxycinnamoyl-coenzyme A: shikimate/quinate hydroxycinnamoyl transferase (*HCT*), hydroxycinnamic acids (HCs), *Aster spathulifolius*, differentially expressed genes (DEGs)

## Abstract

The phenylpropanoid pathway is a major secondary metabolite pathway that helps plants overcome biotic and abiotic stress and produces various byproducts that promote human health. Its byproduct caffeoylquinic acid is a soluble phenolic compound present in many angiosperms. Hydroxycinnamate-CoA shikimate/quinate transferase is a significant enzyme that plays a role in accumulating CQA biosynthesis. This study analyzed transcriptome-wide identification of the phenylpropanoid to caffeoylquinic acid biosynthesis candidate genes in *A. spathulifolius* flowers and leaves. Transcriptomic analyses of the flowers and leaves showed a differential expression of the PPP and CQA biosynthesis regulated unigenes. An analysis of PPP-captive unigenes revealed a major duplication in the following genes: *PAL*, 120 unigenes in leaves and 76 in flowers; *C3′H*, 169 unigenes in leaves and 140 in flowers; *4CL*, 41 unigenes in leaves and 27 in flowers; and *C4H*, 12 unigenes in leaves and 4 in flowers. The phylogenetic analysis revealed 82 BAHDs superfamily members in leaves and 72 in flowers, among which five unigenes encode for *HQT* and three for *HCT*. The three *HQT* are common to both leaves and flowers, whereas the two *HQT* were specialized for leaves. The pattern of *HQT* synthesis was upregulated in flowers, whereas *HCT* was expressed strongly in the leaves of *A. spathulifolius*. Overall, *4CL*, *C4H*, and *HQT* are expressed strongly in flowers and CAA and *HCT* show more expression in leaves. As a result, the quantification of *HQT* and *HCT* indicates that CQA biosynthesis is more abundant in the flowers and synthesis of caffeic acid in the leaves of *A. spathulifolius*.

## 1. Introduction

**Figure 1:**
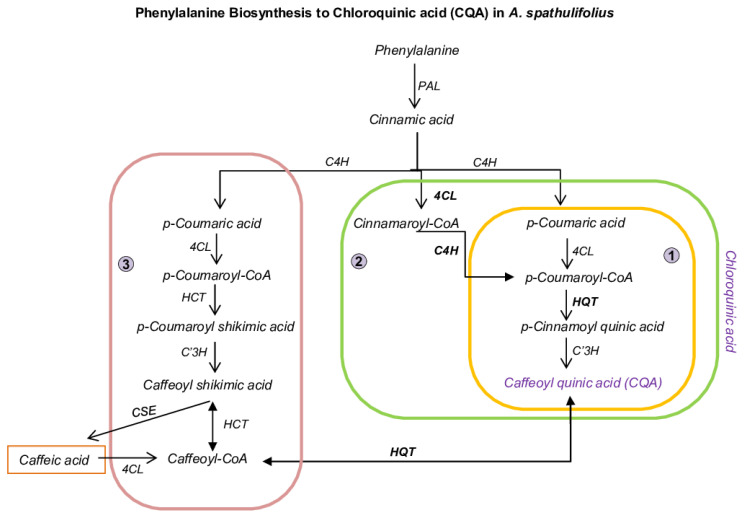
Schematic diagram of phenylalanine towards to caffeoylquinic acid (CQA) biosynthesis in *A. spathulifolius*. 1, 2, and 3 indicate the different route pathways of chlorogenic acid production. ***PAL***, phenylalanine ammonia-lyase; ***C4H***, cinnamic acid 4-hydroxylase; ***4CL***, 4-coumarate--CoA ligase; ***C3′H***, p-coumarate 3′Hydroxylases; ***CSE***, caffeoylshikimate esterase. ***HCT***, hydroxycinnamoyl CoA shikimate hydroxycinnamoyl-transferase; ***HQT***, hydroxycinnamoyl CoA quinate hydroxycinnamoyl-transferase.

Plant secondary metabolites (PSM) are a group of organic compounds that assist in protecting plants against biotic and abiotic stress [1,2,3,4]. PSM are nonessential for plant growth but impart a crucial role as a response to stressful environments [5,6]. Secondary metabolites can be divided into three types: terpenoids, polyketides, and phenylpropanoid [7,8]. The phenylpropanoid pathway (PPP)—the production of enormous compounds by the intermediate process of the shikimate pathway—is present in bacteria, fungi, and plants but absent in animals [9]. The shikimate pathway network connects the carbon metabolism and the AAA (aromatic amino acid) by converting phosphate phenol pyruvate and erythrose-4-phosphate in the glycolysis pentose phosphate pathway to make chorismite, and it synthesizes phenylalanine (Phe) and tyrosine [10,11]. PP metabolites are highly involved in many aspects of plant development and morphological support in response to both biotic and abiotic stress conditions [12]. The main compounds of phenylpropanoid metabolites products of phenylalanine are precursors that regulate many metabolites, such as flavonoids, tannins, lignins, and phenylpropanoid (PP) [11]. These phenolic compounds that produced by PPP assist in the plant defense system against insects and fungi [13]. Phe is the main compound that regulates the phenylpropanoid pathway [14]. Phe helps derive the byproducts of phenol compounds in stress environments and is involved in the production of isoflavonoids, especially in diseased plants; flavonoids in UV irradiation during symbiosis; and salicylic acid in plant-pathogen interactions [15,16]. Caffeoylquinic acid (CQA) or chlorogenic acid (CGA) is the one of the products derived through PPP. CQA has biological effects in blood circulation; its main function is to inhibit the oxidation of low-density lipoprotein in vitro; these assist in human diets [17,18,19]. In a human diet, the biological characteristics of CQA are determined by both its absorption in the gut and its metabolism and are known to be digested by the intestinal microbiota into caffeic acid and quinic acid that have an antiobesity property, which improves lipid metabolism in mice, and antioxidant properties [19,20,21,22,23,24]. These CQA compounds accumulate to substantial levels in coffee, apples, plums, and pears [25,26,27,28]. Previous studies evaluated many Asteraceae species for PP to CQA derived by products in different tissues of plants, and in particular, the sunflower family has been studied widely for CQA production in sprouts, leaves, and roots [29,30,31,32]. During the caffeoylquinic or chlorogenic acid pathway, the enzymes phenylalanine ammonia-lyase (PAL), cinnamate-4-hydroxylase (*C4H*), and 4-coumarate:coenzymeA ligase (*4CL*) are involved in the first steps of the pathway, which are responsible for the synthesis of p-coumarate-CoA. This compound serves as a substrate for CQA synthesis, which is catalyzed by shikimate or quinate acyltransferase and p-coumaroyl ester 3′-hydroxylase (C3′H) (Figure 1). Through PPP to the caffeoyl-quinic acid pathway, the hydroxycinnamoyl-CoA:quinate hydroxycinnamoyl transferase (HQT) played role and was considered to be responsible for 5-caffeoylquinic acid (5-CQA) biosynthesis in coffee, tomato, and potato by transesterification of caffeoyl-CoA and quinic acid; in contrast, tomato and carrot CQA synthesis was discovered by 3′-hydroxylation of p-coumaroyl quinic acid (one of the quinic acids) [33,34]. Unlike shikimic acid, quinic acid is rarely regulated in lignin biosynthesis but is involved in biotic and abiotic stresses, particularly UV radiation [16]. There are numerous hydroxycinnamic acids (HCs) that help in the synthesis various cinnamic acid and 2,3 and 5-CQA, and these major class phenolic compounds are found in Solanaceae, Asteraceae, and Rosaceae [35,36,37]. HCs are part of the acyl-coenzyme A (CoA)-dependent BAHD acyltransferases family; all of these phenolamide production enzymes represent biochemically characterized enzymes: benzylalcohol O-acetyltransferase, anthocyanin O-HCT, HCT of anthranilate, and deacetylvindoline 4-O-acetyltransferase (BAHD-ATs) [38]. Various organic compounds of HCs are based on various types of BAHDs proteins that abundant in apples, peaches, berry, carrot, and coriander, and these compounds are chemically unstable, degradable, and form other compounds, such as flavonoids, isoflavonoids, coumarin, anthocyanins, and lignins [39,40]. The derived byproduct of HCs protects against degenerative and age-related diseases in animals [41,42,43,44]. Most major soluble phenolic (HCs) compounds, such as caffeic acid, coumaric acid, ferulic acid, and sinapic acid, can be found in the Solanum species, such as eggplant, potato, and tomato [45,46,47]. In the caffeoyl-quinic acid synthesis pathway, the effects of adding a combination of hydroxycinnamoyl-coenzyme A: shikimate hydroxycinnamoyl transferase (*HCT)* or hydroxycinnamoyl-coenzyme A: quinate hydroxycinnamoyl transferase (*HQT*) to *C3′H* helps to catalyze the H-unit of p-Coumaroyl CoA into caffeoyl CoA. This process decides the expression and production of caffeoylquinic acid or caffeic acid (HCT) byproducts in plants [35,48,49,50,51]. *HCT* and *HQT* are part of the BAHD acyltransferase superfamily. The *HCT* enzymes use coenzyme A-activated acyl donors, and *HQT* uses quinate as an acceptor, rather than shikimate acid [38]. *HQT* is estimated to be one of the precursors for the synthesis of CQA in most plants [50,52]. A previous study has connected CQA content to PPP and BAHDs (HCT and HQT) gene expression levels. Therefore, in this study, transcriptome and quantitative PCR were used to analyze the CQA biosynthesis response unique to genes in the leaves and flowers of *A. spathulifolius*. The differentially expressed unigenes of PPP throughout the leaf and flower transcriptome were analyzed. Furthermore, using phylogenetic analysis, we analyzed the BAHDs enzyme members consisting of the conserved domain “HXXXD” which represents most CQA involved genes, such as *HQT* and *HCT*. Finally, their putative PPP and BAHDs unigenes were predicted, and candidate unique genes were quantified in different plant parts of *A. spathulifolius*. The identification of PPP and BAHDs provides valuable insights for elucidating the response of metabolites of caffeoylquinic acid biosynthesis in *A. spathulifolius* and an important source of ornamental plants for useful drug discovery.

## 2. Results

### 2.1. Assembly and Gene Annotation

The assembled flower reads produced 146,337 unigenes, with an average contig of 811.58 bp in length. The assembled bases of transcripts had an N50 value of 1279 bp in length and overall alignment rates of 91.71% to the flower raw reads. The GC content was 38.09% on average. In contrast, the leaf transcriptome was reported previously [53]. The assembly completeness was measured by BUSCO analysis to be 91.4% (leaf) and 91.7% (flower), which are the complete transcripts to the eudicots database via tblastn aligns (Figure S1), indicating the good quality of unique transcripts. The flower of *A. spathulifolius* retrieved using the Nr database was 65,129 and 48,896 against the KEGG database, and 70,019 unigenes were identified in the Pfam database (Table 1). The unigenes involved in PPP were found to be 1128 in flower and 1287 in leaf (Table 1). The gene ontology terms of the unique genes of PPP attribute 40.9% to the phenylpropanoid metabolic process, 31.8% to the response to wounding, 22.7% to the lignin biosynthetic process, and 18.2% to the cinnamic acid biosynthetic process in the biological process functions in the *A*. *spathulifolius* transcriptome (Table 2).

### 2.2. Functional Characterization and DEGs of PP Biosynthesis

**Figure 2:**
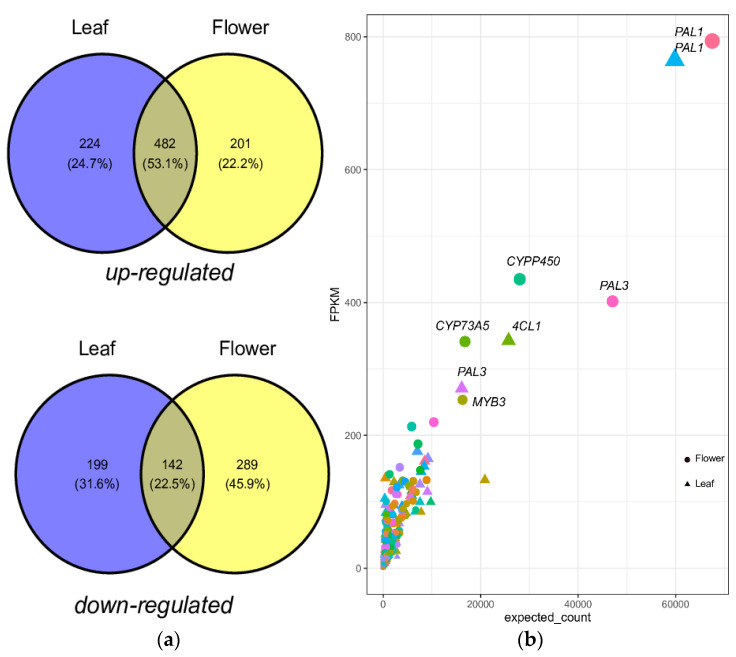
(**a**) A Venn diagram shows up and downregulation of PPP in *A. spathulifolius* leaf and flower. (**b**) The geom point plot shows the upregulated differential expressed genes of PPP in leaf and flower of *A. spathulifolius*.

The unique transcripts of phenylpropanoid (PP) biosynthesis were annotated using KEGG to explore the CQA synthesis pathway in *A. spathulifolius*. The perspective diagram of the CQA biosynthesis pathway in *A. spathulifolius* revealed three different possible routes through the leaf and flower transcriptome analysis: (i) coumaric acid to p-cinnamoyl quinic acid (*p-CQA*) to *C3′H*, (ii) cinnamoyl-CoA with *C4H* followed by p-CQA, and (iii) p-coumaric acid + *4CL* + caffeoyl shikimic acid to caffeoyl CoA with *HQT* to form CQA (Figure 1). From the Venn diagram, it was found that 224 and 201 unigenes were upregulated, and 199 and 289 unigenes were downregulated, specifically in the leaf and flower, respectively. It was also noted that a set of 482 upregulated unigenes and 142 downregulated unigenes indicated the mutual differentially expressed genes between the leaf and flower (Figure 2a, Table S1). Regarding the DEGs unigenes, the *PAL* showed considerable duplication of the genes in both cases of the leaf with 120 unigenes, and the flower had 76 copies of *PAL* genes. *PAL1* was highly regulated in the flower with a 793.68 FPKM value, whereas the leaf showed 765.03 FPKM. The last step to the synthesis of the CQA was the cytochrome P450 (*C3’H*), which catalyzes the 3’-hydroxylation of *p*-coumaric esters of shikimic/quinic acids to form CQA. *C3′H* had 169 unigenes in the leaf and 140 unigenes in the flower of *A. spathulifolius*. There were 27 unigenes in the flower and 41 in the leaf with regard to the *4CL* enzymes. The two *4CL1* genes in the flower were highly regulated with 435~253.48 compared with the leaf. The two leaf *4CL1* with 332.62 and 165 FPKM were observed. *C4H* protein was found to have 12 unigenes in the leaves and 4 unigenes in the flower. The four unigenes of *C4H* were highly upregulated in the leaves, while three unigenes of *C4H* were upregulated in the flowers with a FPKM value of 160~77 in range (Figure 2b, Table S1).

### 2.3. Prediction of BAHDs Superfamily

**Figure 3:**
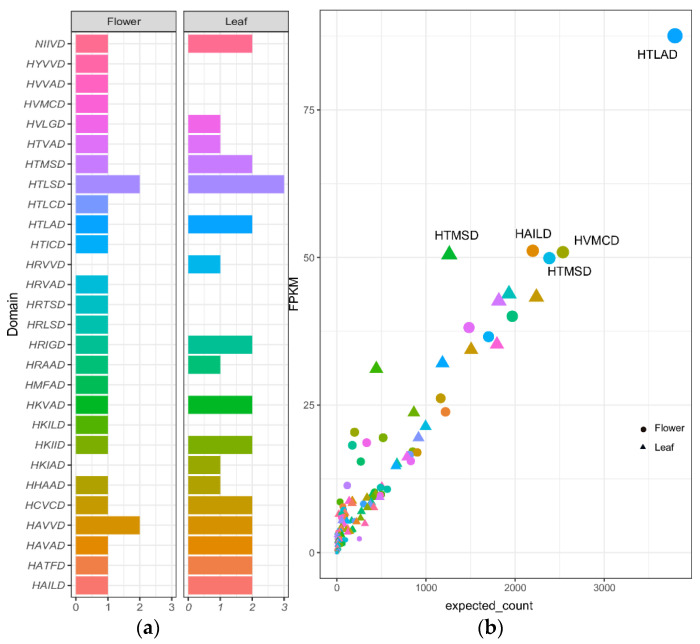
(**a**) Distribution of ’HXXXD’ domain among the leaf and flower of *A. spathulifolius*. The blank space indicates absence of domain; (**b**) The DEGs expression of up and downregulated BAHDs unigenes among the different transcripts.

The prediction of BAHDs superfamily genes was vastly duplicated in *A*. *spathulifolius*. In leaves, there are 704 upregulated PPP unigenes; 82 genes were BAHDs member unigenes among the upregulated unigenes. In contrast, 457 unigenes were found in the flower; 72 unigenes belonged to the BAHD clades, which were upregulated. Among the duplicated BAHDs genes in the flowers with 82 unigenes, 30 of which had ‘HXXXD’ and ‘DFGWG’, two conserved domains were observed. In the leaf, 72 unigenes were related to the BAHDs genes, in that 33 of them had both conserved domains. HTLAD and HTLSD enhance the production of CQA in the chlorogenic acid pathway. The HXXXD domain recognizes the distribution of different BAHDs complex genes; the most available domain was ‘HTLSD’, with five copies in the leaf and three unigenes in the flower, which is involved in the production of the CQA pathway (Figure 3a). The duplication of these unigenes showed different FPKM values with one another in both the leaf and flower. In addition, the distributed domain varied from the flower to leaf; the flower HXXXD domain maintained different amino acid codons than the leaf; even though the leaf has an equal amount of the HXXXD domain, it contains a duplication of the distributed domain. Interestingly, HYVVD, HVVAD, HVMCD, HRVVD, and HKIAD, which were unique to the flower only, are not present in leaf. The domain of HAVVD contains two copies in both the leaf and flower. The HRIGD, HKIID, HAVAD, HATFD, and HAILD domains presented one copy in flowers, whereas they had two copies each in the leaves. The HTLAD (*HQT*)-related unigenes tended to have three duplication genes in the leaf, whereas the flower showed only two copies (Figure 3a). In the flower, the HTLSD (*HQT*) genes were upregulated according to DEGs analysis compared to the leaf *HQT*. The *HQT* domain of ‘HTLAD’ unigenes in the flower expressed strongly, with an FPKM value of 87.58. The HAMSD domain genes with 50.48 were detected in the leaf, respectively. The unknown function of the HTMSD domain genes in both the leaf and flower was strongly expressed in the same value of FPKM (>50) in *A. spathulifolius* (Figure 3b). The lowest expressed domain was the single duplication copy of the HRXXD domain of the HCs unigenes that were identified. Most of the BAHD transferase protein between the flower and leaf showed the highest FRKM value from 87.58 to the lowest of <2.

### 2.4. BAHDs Protein Phylogenies

**Figure 4:**
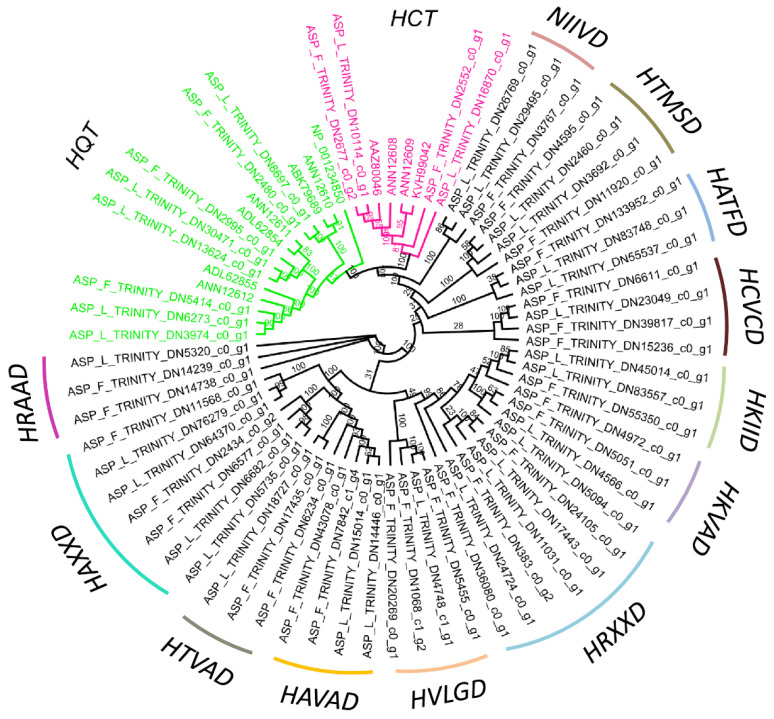
Phylogenetic tree shows the different type of upregulated HXXXD among leaf and flower of *A. spathulifolius*.

The unrooted phylogenetic trees were constructed from a complete ORF and with the presence of the binding site, the ‘HXXXD’ and ‘DFGWG’-conserved domain of the BAHD family members. The HTLAD and HTLSD domains of unigenes were grouped into the previously reported *HQT* genes. The HHAAD domain of *A. spathulifolius* leaf and flower was grouped into the *HCT* unigenes previously reported in other known Asteraceae. Interestingly, the domains of HYVVD and HVVAD also clade within the *HQT* unigenes domains of HTLAD and HTLSD, which are highly diverged in the domain sequence (Figure 4, Figure S2). In contrast, the domain of NIIVD showed the lowest number of duplications in comparison to other distributed BAHDs. The HKXXD-distributed genes were grouped into one clade, showing that the HKIID and HKVAD are highly diverged duplicated BAHDs family proteins with seven copies, even though HKIAD was grouped into another clade after the HRTSD domain. In contrast, most of the HAXXD-based domains were grouped into a single clade with seven duplicated unigenes. Among them, the HRAAD domains showed the least diverged group of genes in *A. spathulifolius*: two copies in the flower and one in the leaf. Although the HTMSD expressed highly in both the leaf and flower, it was missing the single amino acids of ‘HTXSD’ (looks similar to the HQT domain), but it forms a separate clade after the NIIVD domain (Figure 4). Based on the phylogenetic tree, the putative HXXXD domain highly diverged and duplicated in both the leaf and flower of *A. spathulifolius* were identified.

### 2.5. Quantification of PPP Unigenes

**Figure 5:**
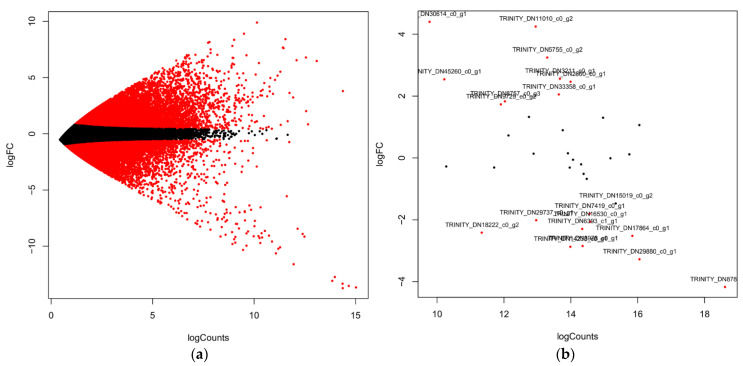
(**a**) MA plot of leaf and flower DEGs in *A. spathulifolius*. Black dot indicates no differentially expression, and below and above red color indicates the down and upregulation of unigenes; (**b**) The plot shows ‘HXXXD’ conserved domain (BAHDs superfamily members), and down and upregulation unigenes in both leaf and flower of *A. spathulifolius*.

**Figure 6:**
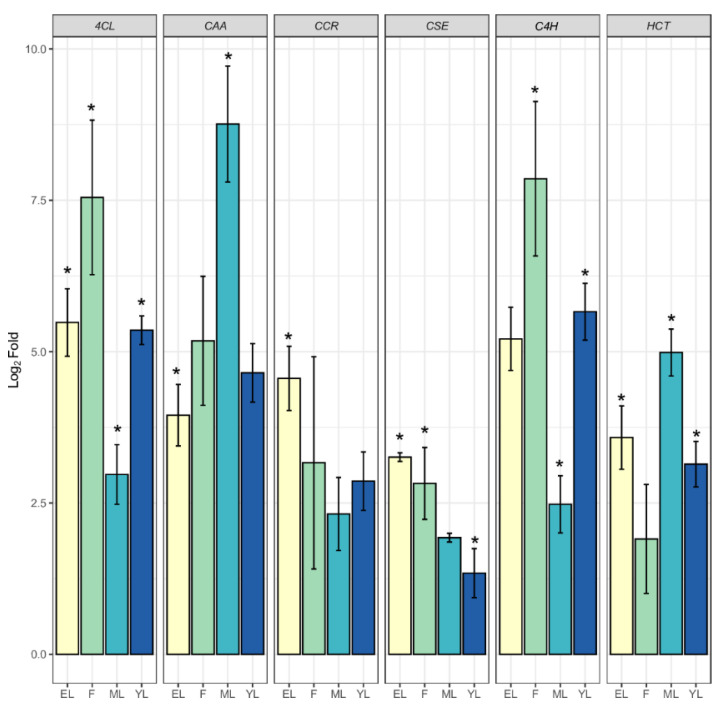
Comparative quantification of PPP unigenes in different plant parts of *A. spathulifolius*. The error bar indicates the mean of ± SD, and * indicates qRT-PCR changes that are statistically significant by t-test (*p* ≤ 0.05). *4CL*; 4-coumarate--CoA ligase, *CAA*; coumaroyl-CoA, *CCR*; cinnamoyl-CoA reductase, *CSE*; caffeoylshikimate esterase; *C4H*, cinnamic acid 4-hydroxylase; *HCT*, hydroxycinnamoyl CoA shikimate hydroxycinnamoyl-transferase.

**Figure 7:**
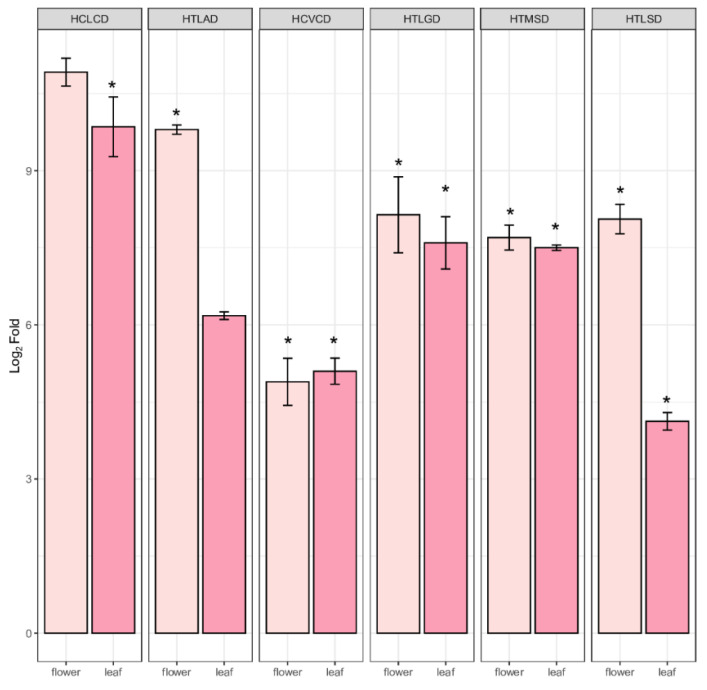
Overlay of quantified expression of HXXXD—hydroxycinnamoyl acyltransferase unigenes in flower and leaf of *A*. *spathulifolius.* The error bar indicates the mean ± SD, and * indicates qRT-PCR changes that are statistically significant by *t*-test (*p* ≤ 0.05). Note: HTLAD and HTLSD belong to HQT class domain.

The combined transcriptome assembled unigenes in both leaf and flower produced 345,781 unigenes. The DEGs analysis of the leaf and flower revealed the up and downregulated unigenes shown in the MA plot. The quantification of DEGs revealed that the top 14,678 were upregulated, and 9789 were downregulated; the remaining unigenes were not differentially expressed and were assigned 0 values (Figure 5a). The flower HCs unigenes quantification revealed the top 9 upregulated genes and 11 downregulated unigenes in the flower, which were also distributed in the leaf transcriptome (Figure 5b). Previous studies on *A*. *spathulifolius* leaf transcriptome-identified PP candidate genes used reverse transcription (RT) to confirm the quality of isoforms produced by assembled transcripts (Figure S3). The production of CQA-derived enzymes in flower and leaf unigenes was identified in the different parts of the leaf, such as young leaf (YL), extended leaf (EL), and mature leaf (ML) stages, which were comparatively quantified (Figure 6). qRT-PCR revealed the high production of *PAL1*, showing high log2fold changes (above >345 FPKM in mature and flower samples), and its quantification increased fourfold in mature and extended leaves; therefore, we removed the PAL1 enzymes from the analysis plot. The *4CL* protein has the highest fold changes in the flower and the second highest activity in the elongation leaf and young leaf. When compared to the elongation leaf and young leaf, *CAA* shows a fold change value of <9.5 in the mature leaf and >5.5 to 4-fold change value in the flower. The *CSE* unigenes were equal to the *4CL* genes, showing a higher Ct fold change value in the flower than in the leaf of *A. spathulifolius*. Finally, the *HCT* had the lowest Ct fold change in the flower, indicating that it was more activated in the leaf rather than the flower. Overall, the flower shows the expression pattern activity of *4CL*, *CAA*, and *C4H*; in contrast, the young leaf shows expression in *4CL*, *HCT*, and *CAA*, and the elongated leaf shows the expression of *4CL* and *CAA* genes (Figure 6). The random six BAHDs gene family was also analyzed using quantitative real-time PCR: HCLCD, HTLAD, HTLSD, HCVCD, HTLGD, and HTMDS, respectively. Quantification of the HCVCD gene showed the lowest expression among the BADHs unigenes chosen. The highest expression was observed in HTLSD with fold changes in flower, and the second highest was observed in unigene HCLCD, followed by the HTLAD, HTMSD, and HTLGD domains of the BAHDs family in the leaf and flower of *A. spathulifolius* (Figure 7). Based on the random ‘HXXXD’ domain, an unknown function of HTMSD expressed highly in both DEGs analysis and qPCR in *Aster*. The significant test indicates that young leaves express the majority of PPP unique genes such as *4CL*, *C4H*, and *HCT*. CAA is significantly expressed in mature and elongated leaves of *A. spathulifolius*. PPP genes such as *4CL*, *CSE*, *C4H*, and HTLAD are significantly expressed and have a high twofold change in amplification in the flower. In the case of putative BAHDs, quantitative expression reveals that the majority of the HXXXD genes are significantly expressed.

## 3. Discussion

Phenylpropanoid biosynthesis produces various secondary metabolites, most of which have beneficial effects on human health [54]. These metabolites are of concern regarding diabetes, obesity, cancer, and cardiovascular disease, which are a significant burden on the world health care system [19,20,21,22,23,24]. CQA is an abundant polyphenol compound in the human diet produced by certain plants, and a variety of mingled pathways have been reported to produce CQA through the phenylpropanoid pathway [20,44,49,52,55]. Coffee has a high concentration of polyphenols (via PPP), which is why coffee has received much attention for CQA production (12, 13 & 14). CQA has an effect on glucose and lipid metabolism, where intermediates control the breakdown of glucose and lipids, [52,55]. Transcriptome and DEGs analysis of *A*. *spathulifolius* revealed an abundance of duplicated PPP unigenes, with the *PAL1* enzyme being the most upregulated when compared to the other *PAL* enzymes. *PAL* catalyzes the conversion of L-Phe to trans-cinnamic acid, which is the gateway to the PPP that leads to various derivative-like flavonoids, isoflavonoids, coumarin, anthocyanins, and lignins [56,57,58]. *PAL* eventually leads to the synthesis of CQA and caffeic acid in plants, which respond differently to biotic or abiotic stresses [11,56,59]. The number of duplicated *PAL* unigenes in leaves and flowers have 120 and 76 copies, respectively, which was more than that found in the Asteraceae species (Cynara with 102 *PAL* genes) [60,61]. The duplication of many *PAL* genes may be important to discuss in the evolution of plant secondary metabolism because these genes are the first step in various secondary metabolisms, and their isoforms contribute different phenolic secondary metabolisms that are downstream of *PAL* [62]. The *4CL* gene was found at its highest levels after the *PAL* enzymes and plays an important role in PPP, belongs to the cytochrome P-450 family. The *4CL* activity results in the higher level of CQA production, and it is also responsible for the synthesis of coumaric acid, ferulic acid, and cinnamic acid [46,63,64]. The duplication of *4CL* may be involved in individual metabolic roles, with distinct metabolic functions, and other important PP biosynthesis activities [63]. The *C3′H* enzyme belongs to the p450 monooxygenases family of the CYP98 family. This enzyme does not use p-coumaric acid as a substrate; it uses the shikimate (*HCT*) and quinic (*HQT*) ester of p-coumaric acid instead. This process catalyzes the 3′-hydroxylation of p-coumaric esters of shikimic/quinic acids to form CQA and synthesizes coniferyl alcohol (G lignins) and sinapyl alcohol (S lignin) [48,55,65]. The p-coumarate 3′hydroxylases (*C3′H)* genes may target the synthesis of caffeoyl CoA to regulate CQAs and other flavonoids [29,32,48]. The genes of *PAL*, *C4H*, *4CL*, *HCT*, and *HQT* are important enzymes, facilitating the biosynthesis of flavonoids and other important secondary metabolites from phenylalanine. The DEGs of HCs (HXXXD) in the leaf and flower show the binding sites of shikimate and quinic acid, which have competition sites to synthesize CQA or caffeic acid via PPP [24,33]. The superfamily of BADHs is a large class of acyl CoA-dependent transferase, which has distinct features and the conserved domains, such HXXXD and DFGWG, consensus sequences, which are highly conserved among plants. These two conserved domains in BAHDs, A C-terminal DFGWG, may or may not be present in all BAHDs, but they provide the structural stability of enzymes. HXXXD has a solid role in catalyzing the acyltransferase group [66,67]. Two *HCT* proteins share the exact conserved domain site of HHAAD in the middle part. The two HXVVD unigenes are closely related to the HCT (HHAAD) proteins, but they do not share the same conserved amino acid as previously studied. They do, however, share other conserved codons with the HCT-like protein, with 78.7% pairwise identity in comparison to the *Cynara* and *Helianthus* (Asteraceae). In contrast, three *HQT*s share the motif of HTLS/AD with *Cynara* and *Helianthus*, with an 87.6% sequence pairwise identity [29,68]. However, there is no conclusive evidence that CQA or caffeic acid is derived from these new HXVVD protein in this research. The *Cynara cardunculus* whole-genome mapping showed that among 32 BAHDs proteins, only three of them showed similarity to the *HCT* and *HQT* enzymes [69]. In vivo studies of CQA synthesis in *C. intybus* revealed two *HCT* and three *HQT* genes involved in *HCT* or *HQT* with *C3′H* to synthesize caffeoyl CoA [68]. The *HCT* and *HQT*-like genes are directly involved in CQA biosynthesis in tobacco, which has also been reported [50]. These type of BAHDs have been extensively studied in Arabidopsis and Populus in terms of an evolutionary relationship, and gene function was found to be involved in plant growth, development, and metabolism [70]. These duplications and new clades of the BAHDs super family may indicate that the PSM is essential to the endurance and successive reproductive fitness of a green plant species in its natural habitat. These results highlight the evolutionary relationships and conserved domain concurrence among the different *HQT* and *HCT* isoforms in the Asteraceae family plants, such as chicory and globe artichoke. On the other hand, the diverged domain of the BAHDs gene complex could have an impact on CQA synthesis in *A. spathulifolius* leaves and flowers. *A*. *spathulifolius* with HTLSD (three) and HTLAD (two) duplicated HQT-like unique genes (in total, five) were expressed differently in the leaf and flower *of A. spathulifolius*. This implies the partition of the CQA synthesis. Along with this, one HCT (HHAAD) and one (HXVVD) have been reported to the HCT-like protein in *A*. *spathulifolius*. Accordingly, both upstream and downstream of the 3-hydroxylation step, HQT and HCT appear to play a significant role in the CQA and caffeic biosynthesis. Aside from HQT and HCT-like proteins, the other newly found HCs may encode for a broad variety of compounds with diverse roles in plant–environment interactions, as well as undiscovered chemical compounds in *A*. *spathulifolius*. This duplication event shows that in *A*. *spathulifolius*, multiple copies of PPP unigenes create a complexity in the process of synthesis of diverse byproducts that has yet to be scientifically examined. Hence, PP biosynthesis depends strongly on an enzyme-dependent method and may accumulate its various metabolites in the flower and leaf of *A. spathulifolius.* This duplication of the core component unigenes suggests that they may have been recruited for major plant phenylpropanoid metabolites for their specialized tissues in *A. spathulifolius*. Based on the transcriptome-wide identification and characterization of CQA in *A. spathulifolius*, the candidate genes involved in the core caffeoylquinic acid synthesis pathway (*PAL*, *C4H*, *4CL, HCT*, *C3′H*, *HQT*) were highly distributed and duplicated throughout the genome. In many metabolic processes, these unigenes are considered important enzymes coding genes. Our results suggest that the HQT-like proteins HTLAD and HTLSD proteins are both expressed differently in the flower and leaf of *A*. *spathulifolius*. In conclusion, our findings targeting *A*. *spathulifolius* have identified a number of PPP and BAHDs family members, in particular HQT-like genes that synthesize caffeoylquinic acid, making them an interesting source for future functional characterization and drug discovery that could benefit human health.

## 4. Materials and Methods

### 4.1. RNA Isolation, cDNA Library Construction, and Illumina Sequencing

The total RNA of the whole flower of *Aster spathulifolius* was extracted using the protocol reported by Bretial et al. [71]. cDNA Library construction and RNA-sequencing were performed using the Genomics Macrogen Laboratory (Korea). The TruSeq method was used to make short fragments of mRNA. The short fragments were then as used as templates for the cDNA library. All short fragments were linked to the sequencing adapter, and the fragments were sequenced for paired-end (PE) reads using illumina sequencing. For leaf transcriptome, the material from previous studies (SRR10724565) [53] and flower RNA-sequencing reads submitted under NCBI-SRA database with accession no: SRR14001926 were used.

### 4.2. Denovo Assembly and Functional Annotation

The raw reads were checked for Fastq quality control [72]. Below quality value ≤ 30% (Q20) reads were removed using the trimmomatic tool [73]. The clean reads were assembled to retrieve unique transcripts using the Trinity program with k-mer size 25 with the following pipeline: inchworm, chrysalis, and butterfly [74]. Finally, the unique reads transcripts were checked for coding function annotations using the ‘Trinotate and TrinotateWeb’ pipeline, as mentioned in a previous study [75]. The gene annotation for all assembled unigenes was aligned to the Swiss-Prot protein database (https://www.uniprot.org/, 31 October 2020), and the Kyoto Encyclopedia of Genes and Genomes (KEGG, http://www.genome.jp/kegg/kegg2.html, 31 October 2020) database. HMMER [76] was used to analyze the complete protein family (Pfam) [77]. The gene ontology was analyzed by the Trinotate pipeline (https://github.com/Trinotate/Trinotate.github.io/wiki, 30 November 2020) and DAVID tools (https://david.ncifcrf.gov/tools.jsp, 30 November 2020) to retrieve the biological process (BP) terms. The quality of the transcripts coding completeness was analyzed using the BUSCO through tblastn alignments against the eudicots database lineage [78].

### 4.3. Identification of PPP Unigenes

The peptide sequence of the Arabidopsis thaliana database of PPP protein was downloaded from the TAIR website [79,80]. The identity of the PPP unigenes was retrieved [72] using local blastX at a value of 1 × 10^5^ using the NCBI-Blast tool [81] and measured against the KEGG database [82] and the BioCyc for pathway prediction (https://biocyc.org/ARA/organism-summary?object=ARA, 31 January 2021). In addition, the PPP involved unigenes of *A. spathulifolius* were set as a database (as a reference database) to blast against the total transcripts to predict the unrecognized sequences for reverification.

### 4.4. Differentially Expressed Genes (DEGs) Analysis

Transcripts of *A. spathulifolius*, differentially expressed genes (DEGs) of the abundance unigenes were determined by RSEM (RNA-seq by expectation-maximization) [83,84], which first generates and processes the total transcripts and then aligns them to raw reads of the *A. spathulifolius* flower and leaf. RSEM is used to calculate the fragments per kilobase per million (FPKM) and the transcripts per million (TPM) values of the total unigenes to analyze the maximum number of genes expressed or abundant in *A. spathulifolius*. The genes were defined as DEGs with the options of FDR-corrected *p* < 0.01 and log2FC > 1. Both FPKM and TPM values were calculated to estimate the expression levels of the unigenes involved in the phenylpropanoid pathway (PPP) and caffeoylquinic acid biosynthesis. Complete DEGs analysis was performed under the Trinity pipeline [74] using RSEM with the option of Bowtie2 [84,85] and EdgeR [86]. The thresholds for considering the significance of DEGs were FDR ≤ 0.01 and |log2 (fold change)| ≥ 1, which is the visualization of results under ggplot2 (https://ggplot2.tidyverse.org/reference/geom_point.html, 31 January 2021).

### 4.5. Structure of BAHD Family Member Unigenes

The protein sequences of BAHD from *Cynara*, *Cichorium* from Asteraceae (same family), and Solanum [50,68,87,88] plants were chosen to identify the *HCT* and *HQT* domain and predict the conserved domain of (HXXXD) of *HCT* and *HQT* under many BAHD family member proteins. The BAHD structural variance was differentiated by aligning the protein sequences by MAFFT [89] with the default parameters and the PHYML (LG) maximum likelihood tree with 100 bootstrap replications [90].

### 4.6. qRT-PCR Expression Studies

Three stages of the leaf (young, extended, and mature leaf), whole flower, and root were used to quantify the CQA biosynthesis-involved unigenes in *A. spathulifolius*. The PP pathway to CQA involving the unigenes from transcriptome leaf data was identified through KEGG annotation and BLAST-p analysis. The unigenes were then searched for the full open reading frame (ORF) to design the forward and reverse primers for further analysis. An amplicon size and primer structure of seven candidate unigenes and random BAHDs unigenes were selected along with the *HQT* genes to quantify (Table S2). cDNA was synthesized by the Reverse Transcription System (A3500, Promega (product made in USA), Yuseong-gu, Daejeon, Korea), and the final 10 µL product was incubated at 70 °C for 5 min to synthesize the c-DNA using Oligo (dT)_15_ Primer. Finally, a quantification assay was carried out using a GoTaq^®^ qPCR Master Mix (A6001, Promega), followed by standard cycling conditions with triplicate as a guideline (Applied Biosystems 7500 step one plus). The significance genes were calculated by *t*-test using R (https://www.rdocumentation.org/packages/stats/versions/3.6.2/topics/t.test, 30 April 2021).

## Figures and Tables

**Table 1:** The statistical comparisons of flower and leaf of *A. spathulifolius* to the annotation of unigenes.

S. No.	Sample	Total Base	Unigenes	Uniprot	KEGG	PPP	BAHD
1	Flower	98,467,589	146,337	65,129	70,019	1128	82
2	leaf	71,660,029	98,860	48,896	39,890	1287	72

**Table 2:** Enriched GO term of biological process (BP) function in *A. spathulifolius* regarding PPP.

GO ID	BP TERM	%	*p*-Value
GO:0009698	phenylpropanoid metabolic process	40.9	9.00 × 10^−21^
GO:0009800	cinnamic acid biosynthetic process	18.2	1.10 × 10^−8^
GO:2000762	regulation of phenylpropanoid metabolic process	18.2	3.80 × 10^−8^
GO:0009611	response to wounding	31.8	4.90 × 10^−8^
GO:0009699	phenylpropanoid biosynthetic process	18.2	6.90 × 10^−6^
GO:0006559	L-phenylalanine catabolic process	13.6	3.10 × 10^−5^
GO:0009809	lignin biosynthetic process	22.7	2.80 × 10^−4^
GO:0008152	metabolic process	13.6	3.80 × 10^−4^
GO:0009411	response to UV	13.6	2.40 × 10^−3^
GO:0080167	response to karrikin	13.6	8.30 × 10^−3^
GO:0009813	flavonoid biosynthetic process	13.6	1.10 × 10^−2^

## Supplementary Materials

The following are available online at https://www.mdpi.com/article/10.3390/ijms22126333/s1.
