# Supplementary material for: Transcriptome-Wide Identification and Quantification of Caffeoylquinic Acid Biosynthesis Pathway and Prediction of Its Putative BAHDs Gene Complex in A. spathulifolius
Source: Int J Mol Sci. 2021 Jun 13;22(12):6333. doi: 10.3390/ijms22126333 (PMC8231772; doi:10.3390/ijms22126333)
Supplement: Supplementary file 1 [file ijms-22-06333-s001.zip › ijms-1249299 supplementary/Supplementary.pdf]

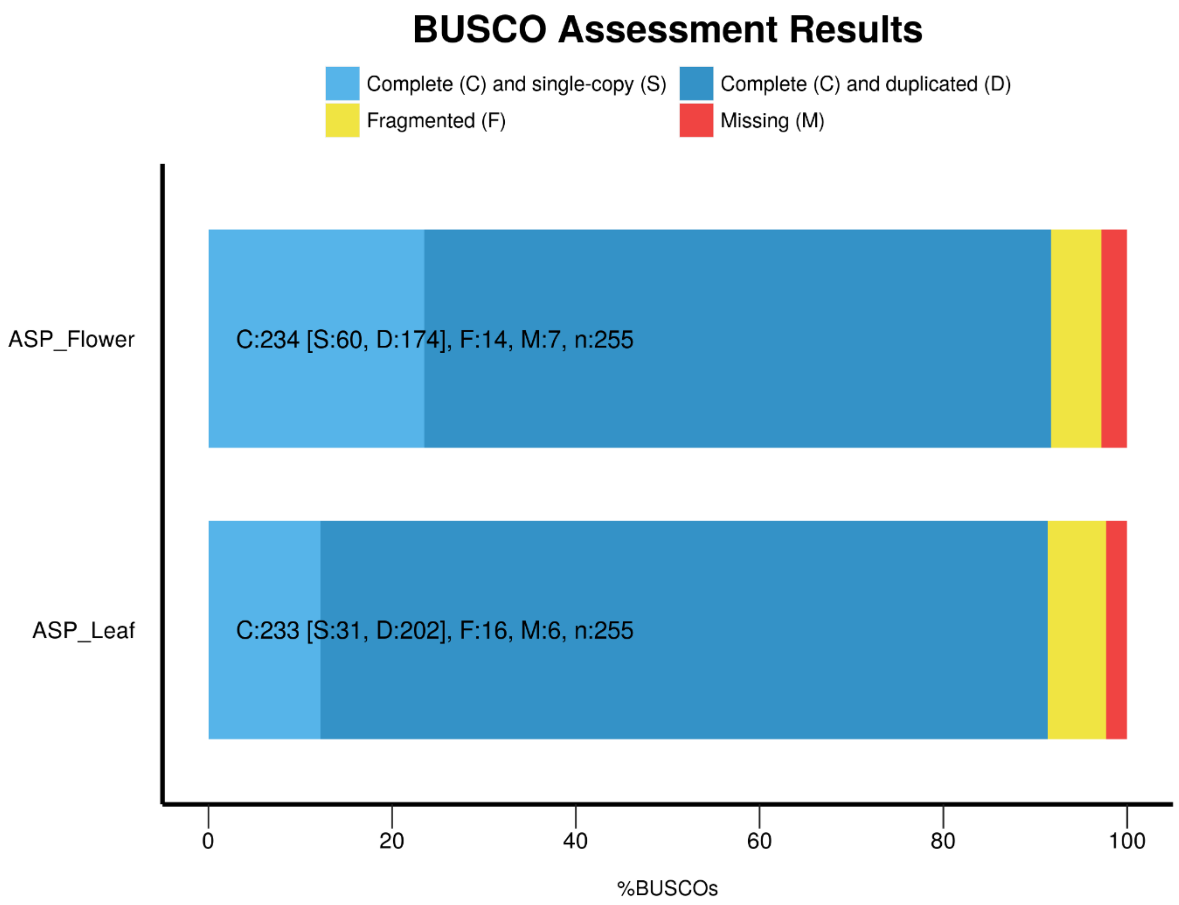

Figure S1. BUSCO assessments shows the quality of transcriptome assembled unigenes. C:complete, D: Duplicated, F: fragments, M: missing

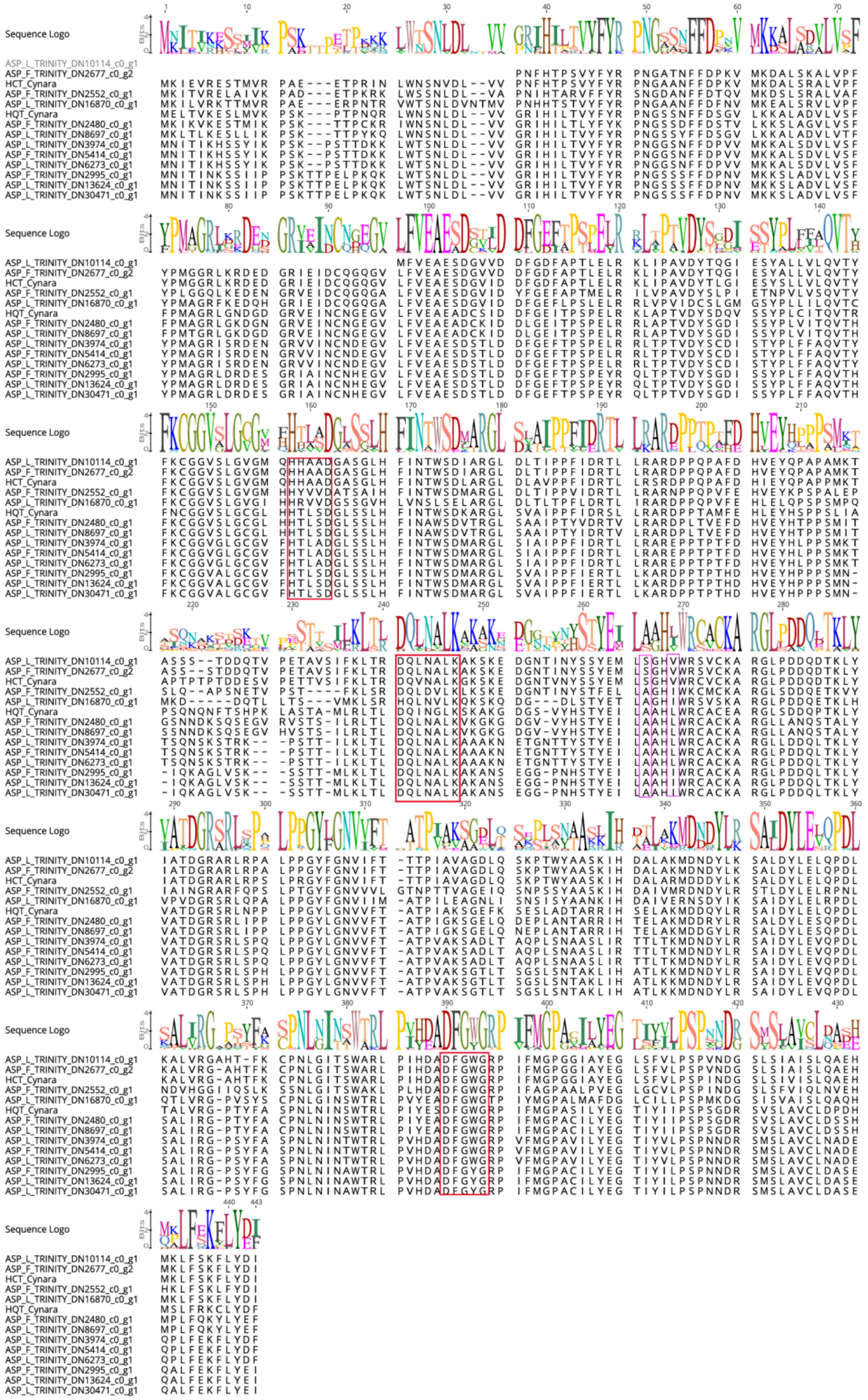

Figure S2: Alignment of HQT and HCT unigenes of *A. spathulifolius* to the *Cynara* species. Red and pink bar indicates the most conserved domain shares between HCT and HQT genes.

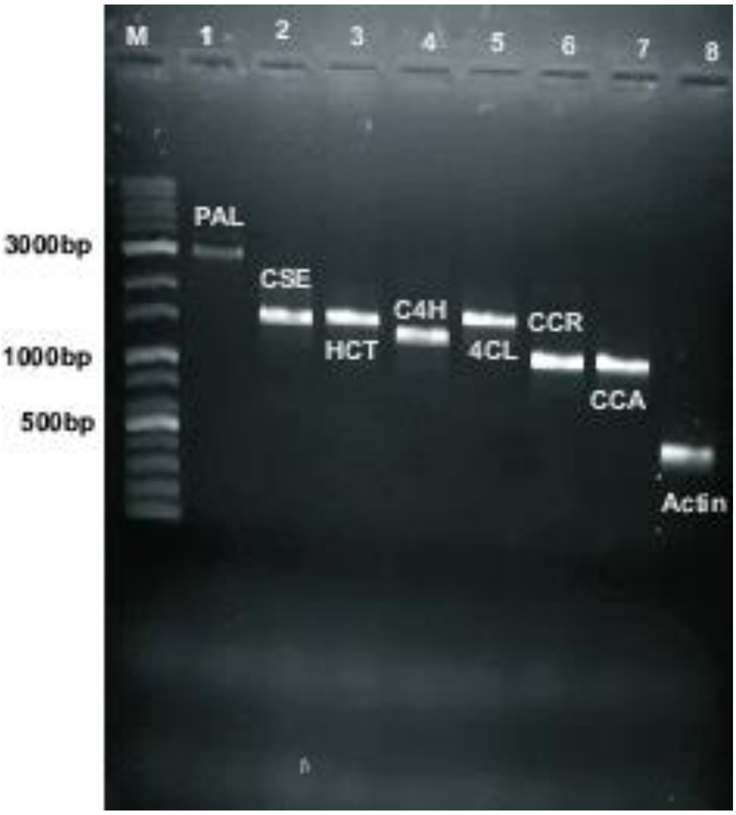

Figure S3 : RT-PCR conformation of PPP unigene in *A. spathulifolius* leaf of *denovo* assembled unigenes.
